# Transactions of the American Dental Association for 1876

**Published:** 1877-10

**Authors:** 


*Transactions of the American Dental Association for* 1876.

This volume contains the proceedings of the centennial meeting held in Philadelphia, and contains some two hundred and thirty pages. We regret, however, that its publication was so long delayed, as it did not appear until nearly the time of the 1877 meeting, had arrived consequently the contents were almost one year old.

---

*Transactions of the Illinois State Dental Society for* 1877.

